# Dual Stiffened Small-Intestinal Diverticuli Compressing the Common Bile duct

**DOI:** 10.51894/001c.165587

**Published:** 2026-07-31

**Authors:** Terrance Duncan, David Neff

**Affiliations:** 1 College of Osteopathic Medicine Michigan State University

## 101

### Introduction

Small-intestinal diverticula are uncommon and usually incidental; when symptomatic, they can produce vague upper-abdominal complaints from stasis, inflammation, or mass effect. Periampullary duodenal diverticula (PAD) are reported in ~3–32% of ERCP cohorts and increase with age, yet most are asymptomatic. Lemmel’s syndrome is obstructive jaundice from extrinsic compression of the distal common bile duct (CBD) by a periampullary diverticulum in the absence of stones or malignancy. Concurrent CBD compression by multiple diverticula is exceptionally rare and described only in isolated cases without a calculable incidence.

### Case Description

A 75-year-old man reported several weeks of right upper-quadrant pain triggered by bending forward and nausea when hungry or soon after meals. He denied fever, jaundice, vomiting, bowel changes, or weight loss and remained afebrile. Laboratory testing was normal, including complete blood count, liver enzymes, alkaline phosphatase, bilirubin, amylase/lipase, erythrocyte sedimentation rate, and C-reactive protein. Ultrasound, contrast-enhanced CT, and MRCP showed two duodenal diverticula with retained debris, including undigested gelatin capsule fragments. Intermittent CBD dilatation appeared on some—but not all—studies. Upper endoscopy was normal, with normal biopsies. Although Lemmel’s syndrome requires obstructive jaundice from extrinsic CBD compression, the intact liver chemistries and absence of jaundice indicate this is not classic Lemmel’s syndrome. Nevertheless, the intermittent CBD dilatation supports partial, positional extrinsic compression, likely exacerbated by stiffness of retained gel-cap fragments, slowing bile drainage and provoking RUQ pain and post-prandial nausea without fever, inflammation, or infection.

### Conclusions

This case highlights an atypical, debris-mediated presentation of small-intestinal diverticula with positional RUQ pain and post-prandial nausea despite normal hepatopancreatic labs and normal biopsies. Multimodal imaging was essential to distinguish partial, intermittent extrinsic compression from biliary obstruction. While PADs are relatively common in older adults, symptomatic biliary compression is rare, and cases with multiple diverticula impinging the CBD are extraordinarily rare. Clinicians should consider debris-laden peri-duodenal diverticula in older adults with unexplained RUQ discomfort and normal laboratory studies when imaging shows intermittent CBD dilatation. A multidisciplinary team approach should be used to monitor and intervene as necessary because surgical complications could be equally high.

